# Screen-time is associated with inattention problems in preschoolers: Results from the CHILD birth cohort study

**DOI:** 10.1371/journal.pone.0213995

**Published:** 2019-04-17

**Authors:** Sukhpreet K. Tamana, Victor Ezeugwu, Joyce Chikuma, Diana L. Lefebvre, Meghan B. Azad, Theo J. Moraes, Padmaja Subbarao, Allan B. Becker, Stuart E. Turvey, Malcolm R. Sears, Bruce D. Dick, Valerie Carson, Carmen Rasmussen, CHILD study Investigators, Jacqueline Pei, Piush J. Mandhane

**Affiliations:** 1 Department of Pediatrics, University of Alberta, Edmonton, Alberta, Canada; 2 Department of Medicine, McMaster University, Hamilton, Ontario, Canada; 3 Department of Pediatrics & Child Health, Children’s Hospital Research Institute of Manitoba, University of Manitoba, Winnipeg, Manitoba, Canada; 4 Department of Pediatrics, Hospital for Sick Children, University of Toronto, Toronto, Ontario, Canada; 5 Department of Pediatrics, Child & Family Research Institute, BC Children’s Hospital, University of British Columbia, Vancouver, British Columbia, Canada; 6 Department of Anesthesiology and Pain Medicine, University of Alberta, Edmonton, Alberta, Canada; 7 Faculty of Physical Education and Recreation, University of Alberta, Edmonton, Alberta, Canada; 8 Department of Educational Psychology, University of Alberta, Edmonton, Alberta, Canada; International Telematic University Uninettuno, ITALY

## Abstract

**Background:**

Pre-school children spend an average of two-hours daily using screens. We examined associations between screen-time on pre-school behavior using data from the Canadian Healthy Infant Longitudinal Development (CHILD) study.

**Methods:**

CHILD participant parents completed the Child Behavior Checklist (CBCL) at five-years of age. Parents reported their child’s total screen-time including gaming and mobile devices. Screen-time was categorized using the recommended threshold of two-hours/day for five-years or one-hour/day for three-years. Multiple linear regression examined associations between screen-time and externalizing behavior (e.g. inattention and aggression). Multiple logistic regression identified characteristics of children at risk for clinically significant externalizing problems (CBCL T-score≥65).

**Results:**

Screen-time was available for over 95% of children (2,322/2,427) with CBCL data. Mean screen-time was 1·4 hours/day (95%CI 1·4, 1·5) at five-years and 1·5 hours/day (95%CI: 1·5, 1·6) at three-years. Compared to children with less than 30-minutes/day screen-time, those watching more than two-hours/day (13·7%) had a 2·2-point increase in externalizing T-score (95%CI: 0·9, 3·5, p≤0·001); a five-fold increased odd for reporting clinically significant externalizing problems (95%CI: 1·0, 25·0, p = 0·05); and were 5·9 times more likely to report clinically significant inattention problems (95%CI: 1·6, 21·5, p = 0·01). Children with a DSM-5 ADHD T-score above the 65 clinical cut-off were considered to have significant ADHD type symptoms (*n* = 24). Children with more than 2-hours of screen-time/day had a 7·7-fold increased risk of meeting criteria for ADHD (95%CI: 1·6, 38·1, *p =* 0·01). There was no significant association between screen-time and aggressive behaviors (*p*>0.05).

**Conclusion:**

Increased screen-time in pre-school is associated with worse inattention problems.

## Introduction

Childhood screen-time has increased over the years[1–4]. Increased screen-time has been associated with unhealthy dietary patterns, poor sleep quality, cardiovascular disease, and obesity[5] in children. In 2016, the new Canadian 24-hour Movement Guidelines[6, 7] recommend that children aged five and over should have less than two-hours of screen-time/day, while limiting screen-time to less than one-hour/day for two-four year olds. It has been estimated that children between three to five years old are exposed to an average of two-hours of screen-time per day[8–13] in Canada.

There has been a significant increase in screen options in recent years, from device choices to streaming content, with rising concern that screen-time may have negative consequences for mental health[14]. Studies of school-aged children have shown associations between increased television viewing time and attention problems[15–17]. The Dunedin Study found that increased television viewing from five to eleven years old was associated with attention problems in adolescence[18]. In their study sample, children reportedly watched an average of 2-hours of television each day between[18]. Recent study examining adolescent sedentary behaviors among suggested that screen-time should be considered a risk factor for attention deficit hyperactivity disorder (ADHD) symptoms[19]. Swing et al[20] reported that television viewing time greater than 2-hours per day was associated with increased attention problems among older children. A meta-analysis concluded that television viewing or gaming among 4–17 years old children was modestly associated with later ADHD symptoms[4]. Reports in toddlers and preschoolers have linked television viewing time above 1.5 hours to later behavioral and emotional problems[8]. There is less research examining associations between screen-time exposure and behavioral development in the preschool years. Most studies have focused on school-aged children, only considered traditional screen sources such as television viewing, or did not allow for many potential confounders.

We analyzed data from the population-based Canadian Healthy Infant Longitudinal Development (CHILD) birth cohort study to determine associations between screen-time and behavioral outcomes at age 5 years. Prolonged screen-time may displace time spent in other activities such as active play; important to promoting development in young children[21, 22]. The extensive CHILD Study assessment allowed us to examine major determinants of mental health including movement behaviors (e.g. screen-time, sleep, physical activity) in addition to important covariates such as parenting stress, socioeconomic status, marital status, and breastfeeding. We investigated whether parents of children exposed to more screen-time reported more externalizing and internalizing behavior problems at 5 years of age. We sought to determine if increased screen-time above the Canadian recommended guideline is associated with clinically significant behavior problems in young children. We hypothesized that children exposed to screen-time above the 2-hour threshold would exhibit clinically significant (T-Score ≥65) ADHD type behavior problems.

## Materials and methods

### Study population and design

This study involved a population-based sample of 3,455 children recruited in four Canadian (Edmonton, Toronto, Vancouver, and Manitoba) from the CHILD study (www.childstudy.ca). CHILD is a naturalistic observational study initially designed to examine gene-environment interactions on the development of asthma and atopy[23, 24]. Pregnant mothers ≥18 were recruited nationally from hospital and birth centres in the second or third trimester. Parent-child dyads were enrolled during pregnancy between 2009 and 2012. Parents completed questionnaires about family and child characteristics (e.g. socioeconomic status (SES), ethnicity), maternal and infant nutrition, and maternal stress at baseline and follow-up. At three and five-year follow-up clinic visits, participating families completed questionnaires about their child’s screen-time, sleep quality, and physical activity. CHILD participants parents completed a questionnaire assessment about their child’s behavior[25] at five-years. Informed consent was obtained from all mothers, and consenting fathers. The study was approved by the University of Alberta Research Ethics Board, the University of British Columbia Research Ethics Board, the University of Manitoba Research Ethics Board, and the University of Toronto Research Ethics Board and The Hospital for Sickkids.

Data on potential covariates associated with screen-time or behavior were obtained from hospital records (gender, birth weight in kg, gestational age in weeks, birth order, sibling status, gestational diabetes, maternal age) or standardized questionnaires (sleep duration or sleep disruption, physical activity, SES, marital status, ethnicity, household smoke exposure, breastfeeding, maternal depression, parent-child interaction, and parenting stress at five years visit). A detailed overview of the covariates included in this analysis is provided in the S2 File.

#### Screen-time (primary exposure variable)

Screen-time (hh:mm) was assessed at ages three and five-years. Parents reported their child’s total screen-time/day, which included watching TV/DVD’s, using a computer, tablet, mobile phone, or playing video games. Screen-time was grouped into three categories based on the recommended Canadian 24-hour Movement Guidelines for children 5–13 years(6): 1) less than 30-minutes/day; 2) between 30-minutes and two-hours daily; or 3) more than 2-hours. The upper threshold for total screen-time at age three-years was adjusted to one-hour per day based on the Canadian 24-hour Movement Guidelines for Young Children[26]. The Canadian guidelines provide screen-time recommendations that are consistent with the American Academy of Pediatrics recommendations[22, 27].

#### Preschool behavior (outcome variable)

The Child Behavior Checklist (CBCL) preschool version[28] was completed by parents at age five-years at all CHILD sites. The CBCL is a 99-item empirically validated measure of parent-reported behavior problems in early childhood[28]. The CBCL produces a T-score (mean of 50 points and a standard deviation of 10 points based on normative data) for *internalizing* problems (e.g. anxious/depressed, withdrawal, somatic, emotionally reactive), *externalizing* problems (e.g. inattention, aggressiveness) and *total problems* (internalizing, externalizing, sleep issues, and other problems)[28, 29]. The CBCL also includes five Diagnostic and Statistical Manual of Mental Disorders (DSM-5)-oriented syndrome scales including attention deficit hyperactivity (ADHD) problems, oppositional defiant problems, depressive problems, anxiety problems, and autism spectrum disorder. The DSM-ADHD scale has good predictive validity of ADHD diagnosis in community and outpatient samples[30]. Higher scores indicate greater behavior problems. We used the externalizing (primary outcome), internalizing (secondary outcome) and total (secondary outcome) linear T-scores for this analysis. We also applied the cut-off score of ≥65 points which indicates clinically significant behavior problems based on the CBCL manual[28].

### Statistical analysis

Children with reported developmental or genetic disorders, such as autism, diagnosed by their healthcare professional, (n = 71) were removed from all analyses. Characteristics of those families who provided CBCL data were compared to those without CBCL data using chi-squared analysis for categorical variables and t-test for continuous variables.

Univariate analyses, *t-test* for dichotomous predictors and linear regression for continuous variables, were used to identify associations between screen-time categories (primary exposure variable), sleep duration, sleep disordered breathing (SDB), physical activity, child and family characteristics and CBCL assessed externalizing (primary outcome), internalizing, and total behavior problems (secondary outcomes) at five-years.

Multiple linear regression (entry method) was used to assess the association between screen-time and behavior problem scores while adjusting for child gender and factors significant in univariate analysis (*p*≤0·05). The final model was determined based on the Akaike Information Criterion (AIC) where the lowest values indicated the best model fit. Missing values for all covariates were replaced with the mean for continuous for variables and the reference for categorical variables. A dummy variable was included in the analysis in order to account for the mean replacement for continuous variables. A sensitivity analysis was conducted to explore associations of movement behaviors known to interact and influence one another in accordance with the 24- Canadian 24-hour Movement Guidelines[6, 7]. Interactions terms for screen-time, physical activity, and sleep were considered in multiple linear regression analyses (*p*’s≤0·05). We completed a sensitivity analysis (multiple logistic regression) to examine the association between screen-time and clinically significant behavior problems using a cut-off CBCL T-score of ≥65. Statistical analysis was completed using STATA, version 14.

## Results

### Sample characteristics

Of the 3,455 families enrolled in the CHILD study, 2,427 (70·2%) children had CBCL data at five-years. Those with CBCL data had a higher family income and were more likely to be Caucasian (see Table 1). Parents of children with CBCL data reported significantly less screen-time/day than those children without CBCL data (Mean 1·4 hours; 95%CI 1·4, 1·5 vs. 1·8 hours; 95%CI 1·5, 2·1 vs. *p =* 0·01; Table 1).

**Table 1:** Demographic characteristics for children with and without CBCL data at five-years of age.

**Categorical**	**Data Absent % (behaviour/total)**	**Data Present % (behaviour/total)**	**p-value**
Boys	52.7% (522/990)	52.3% (1268/2427)	0.80
Child ethnicity: Caucasian	58.9% (575/977)	68.8% (1606/2403)	≤0.001
Annual income > $60, 000	70.2% (571/813)	86.1% (1986/2307)	≤0.001
Marital divorce or separation at 5 years	12.5% (4/32)	6.6% (137/2076)	0.19
Attend preschool at 5 years: yes	78.6% (33/42)	69.9% (1402/2006)	0.32
Sleep disordered breathing symptoms: yes	13.4 (15/112)	8.4% (185/2214)	0.66
**Continuous**	**Data Absent** ***Mean* (95%CI)**	**Data Present** ***Mean* (95%CI)**	**p-value**
Maternal age at time of child’s birth	31.6 (31.2, 31.9)	32.6 (32.4, 32.8)	≤0.001
Screen-time at 5 years	1.8 (1.5, 2.1)	1.5 (1.4, 1.5)	0.01
Hours spent in organized physical activity	3.2 (2.5, 3.9)	2.6 (2.5, 2.7)	0.11
Total sleep duration (hours)	10.9 (10.7, 11.0)	10.9 (10.9, 11.0)	0.42

Caption: Population characteristics based on Chi-square analysis for categorical variables and t-test for continuous variables.

### Prevalence of behavior problems at age five-years

Clinically significant externalizing behavior problems (T-score ≥65) was observed among 1·2% of children (*n* = 28), while 2·5% of children (*n* = 61) exhibited clinically significant internalizing behavior problems. Less than 1% of children (*n* = 18) had both clinically significant internalizing and externalizing behavior problems.

Boys had a higher CBCL externalizing T-score (mean = 40·6, *SD* = 9·9) than girls (mean = 38·7, *SD* = 9·0; *p =* ≤0·001). Boys were more likely to be classified as having clinically significant (T-score ≥65) externalizing behavior problems than girls (*n* = 41 vs *n* = 20, *p*≤0·05). There were no differences by gender observed for internalizing behavior problems.

### Exposure to screen-time

**Fig 1:**
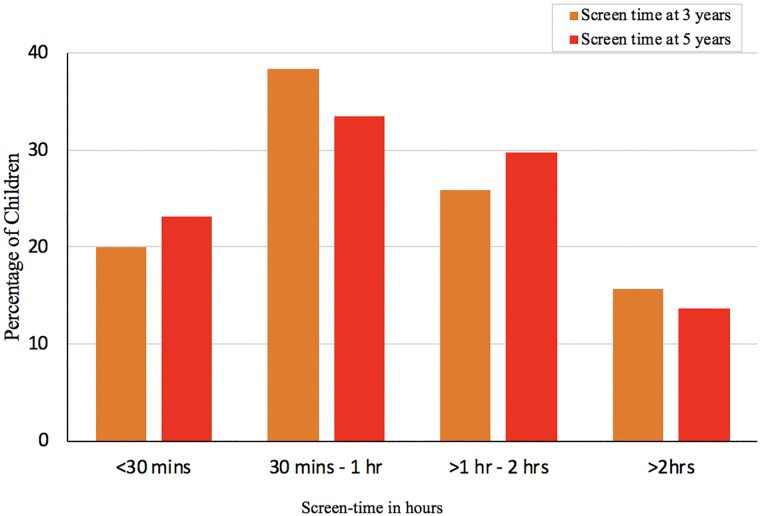
Amount of screen-time in hours/day reported by parents at three and five-years.

Screen-time data was available for 96% (2322/2427) of participants whose parents had completed the CBCL questionnaire. At five-years, over 13% (*n =* 317) of children were exposed to more than 2-hours of screen-time/day while 83% (*n =* 2,005/2,427) of children met the Canadian recommended[22] screen-time guidelines of less than 2-hours per day (Fig 1). At three-years, 58% of children (1,415/2,427) met the Canadian recommended screen-time guideline of less than 1-hour of screen-time/day. There were no significant differences by gender for reported screen-time.

### Children exposed to more screen-time showed increased behavioral morbidity

In a dose-response manner, children exposed to more screen-time, at either age three and five-years, showed significantly increased behavior problems at five-years in univariate analysis. Briefly, children who watched more than 2-hours of screen-time/day had increased *externalizing*, *internalizing*, *and total* behavior problems scores compared to children who watched less than 30-minutes. Univariate linear results for the CBCL composite scales are provided in S1 Table through S9 Table.

Screen time remained significant in the linear multivariate results for total, internalizing, and externalizing behavior. Adjusted results for total behavior problems (secondary outcome) are presented in S3 File and S9 Table.

#### CBCL externalizing behaviour multiple linear regression analyses (primary outcome)

Children exposed to more than 2-hours of screen-time had a 2·2-point increase in externalizing behavior problem T-score (95%CI: 0·9, 3·5, *p*≤0·001; model 1, Table 2) when controlling for sleep duration, parent-reported SDB symptoms, gender, SES, marital status, parenting stress, and maternal depression. Children who spent two or more hours/week participating in organized physical activities showed a 1·3-point reduction in externalizing behavior score (95%CI: -2·1, -0·6, *p*≤0·001). There were no significant interactions between screen-time and gender, physical activity, school enrolled, sleep duration, SDB, or parenting stress (all *p*>0·05). The association between screen-time at five-years and increased externalizing behavioral morbidity remained significant when controlling for daily reported hours of screen-time at three-years (*p*≤0·05).

**Table 2:** Multiple regression analysis examining associations between screen-time and the CBCL externalizing behavior problem T-score at five-years of age (n = 2,427).

	Model 1: Linear score Externalizing T-score		Model 2: Clinical cut-off Externalizing T-score ≥65	
Factor	Coefficient 95%CI	p-value	Odds Ratio 95%CI	p-value
**Screen-time at 5 years**: Reference: < 30-minutes daily	Reference		Reference	
Between 30-minutes and 2 hours daily	0.6 (-0.3, 1.5)	0.21	2.3 (0.5, 10.4)	0.28
More than 2 hours daily	2.2 (0.9, 3.5)	≤0.001	5.0 (1.0, 25.0)	0.05
**Organized physical activity at 5 years**: More than 2 hours/week: yes	-1.3 (-2.1, -0.6)	≤0.001	-	-
**Parent-reported SDB symptoms at 5 years**: Yes	1.7 (0.4, 3.0)	0.01	-	-
**Parent-reported total sleep duration less than 10 hours/24-hour at age 5**: yes	0.5 (-0.5, 1.6)	0.35	2.4 (1.0, 5.5)	0.04
**Gender**: male	2.0 (1.3, 2.7)	≤0.001	5.1 (1.9, 14.1)	≤0.001
**SES: Family income** ≥ **$60,000 income** (reference: < $60,000 / year)	-1.8 (-2.9, -0.6)	≤0.001	0.2 (0.1, 0.5)	≤0.001
**Marital status at 5 years**: separated, divorced, or widowed: yes (reference: married or common law)	2.8 (1.1, 4.6)	≤0.001	-	-
**Gestational diabetes**: Yes	2.3 (0.0, 4.6)	0.05	4.8 (1.2, 19.3)	0.03
**Parenting stress at age 5 using the PSI-SF Scale**	0.3 (0.2, 0.4)	≤0.001	1.2 (1.1, 1.3)	≤0.001
**Maternal depression at age 5 using the CES-D Scale**	0.2 (0.1, 0.2)	≤0.001	-	-

Caption: SDB = Sleep Disordered Breathing, based on 6 items; PSI-SF = Parenting Stress Index-Self Report, higher score presents increased levels of parenting stress; CES-D = Centre for Epidemiological Studies—Depression, higher scores represent increased maternal symptoms of depression

Clinically significant externalizing behaviors (CBCL≥65): Parents of children exposed to more than two-hours of screen-time were 5-times more likely to report clinically relevant externalizing behavior problems (OR 5·0, 95%CI: 1·0, 25·0, *p* = 0·05; model 2, Table 2) compared to parents of children exposed to less than 30-minutes of screen-time/day. Aside from gender, screen-time had the strongest association with externalizing behavioral morbidity. Additional covariates associated with externalizing behavior included shorter sleep duration, SES, and parenting stress (Table 2). There was no significant association between physical activity, SDB, marital status, or maternal depression and externalizing behavior problems in adjusted analyses.

#### Externalizing behavior subscales

We explored the association between screen-time and the inattention and aggressive externalizing behavior subscales in multiple linear regression analysis. Screen-time above the 2-hours threshold was significantly associated with an inattention problem score above the clinical cut-off of 65 (5·9 OR, 95%CI: 1·6, 21·5, *p =* 0·01; model 1, Table 3), compared to children with less than 30-minutes of screen-time per day. That is, parents of children exposed to more screen time were 5.9 times more likely to report inattention behavioral morbidity. There was no significant association between screen-time and aggressive behavior problems (model 2, Table 3).

**Table 3:** Logistic multiple regression analysis examining associations between screen-time and the CBCL attention problems and aggression subscales at five-years of age (n = 2,427).

	Attention Problems Clinical cut-off ≥65		Aggression Clinical cut-off ≥65	
Factor	Coefficient 95%CI	p-value	Coefficient 95%CI	p-value
**Screen-time at 5 years of age**: Reference: < 30-minutes daily	Reference		Reference	
Between 30-minutes and 2 hours daily	3.0 (0.9, 10.2)	0.07	1.9 (0.6, 6.8)	0.06
More than 2-hours daily	5.9 (1.6, 21.5)	0.01	2.1 (0.5, 8.6)	0.31
**Organized physical activity at age 5**: More than 2-hours/week: yes	-	-	0.2 (0.1, 0.8)	0.02
**Parent-reported total sleep duration less than 10 hours/24-hourat age 5**: Yes (reference > 10 hours/night)	-	-	2.3 (1.0, 5.2)	0.04
**Gender**: male	3.9 (1.9, 7.9)	≤0.001	-	-
**SES: Family income ≥ $60,000 income** (reference: < $60,000 / year)	0.4 (0.2, 0.8)	0.01	0.2 (0.1, 0.5)	≤0.001
**Marital status**: separated, divorced, or widowed: yes (reference: married or common law)	-	-	-	-
**Parenting stress at age 5 using the PSI-SF Scale**	1.1 (1.1, 1.2)	≤0.001	1.2 (1.1, 1.2)	≤0.001
Constant	0.2^2^ (0.3^3^, 0.9^2^)	≤0.001	0.5^2^ (0.9^3^, 0.3^1^)	≤0.001

Caption: SES = Socio-economic status; PSI-SF = Parenting Stress Index-Self Report, higher score presents increased levels of parenting stress

Children with a DSM-5 ADHD T-score above the clinical cut-off of 65 were considered to have significant ADHD type symptoms (*n* = 24). More than 2-hours of screen-time/day was significantly associated with an ADHD score above the clinical-cut-off of 65 (7·7 OR, 95%CI: 1·6, 38·1, *p =* 0·01; Table 4, model 2) adjusting for gender, gestational diabetes, and parenting stress. Suggesting that parents of children exposed to excessive screen-time were more likely to report ADHD type behavioral morbidity. The effects remained significant even after removing from our analyses five children who had been previously diagnosed with ADHD by a healthcare professional.

**Table 4:** Multiple logistic regression analysis for ADHD morbidity using the CBCL Diagnostic Statistical Manual (DSM-5) oriented scale (n = 2,427).

	Model 1: Linear score DSM-5 ADHD T-score		Model 2: Clinical cut-off DSM-5 ADHD T-score ≥65	
Explanatory variable	Coefficient 95%CI	p-value	Odds Ratio 95%CI	p-value
Screen-time at 5 years: Reference: < 30-minutes daily	Reference		Reference	
Between 30-minutes and 2 hours daily	0.2 (0.0, 0.5)	0.10	1.9 (0.4, 8.7)	0.43
More than 2-hours daily	0.8 (0.4, 1.2)	≤0.001	7.7 (1.6, 38.1)	0.01
Organized physical activity at age 5 years: More than 2-hours/week: yes	-0.3 (-0.6, -0.1)	0.01	-	-
Parent-reported SDB symptoms at age 5 years: Yes	0.6 (0.2, 1.0)	≤0.001	-	-
Gender: Male	0.5 (0.3, 0.7)	≤0.001	7.4 (2.2, 25.4)	≤0.001
SES: Family income ≥ $60,000 income (reference: less than $60,000/year)	-0.5 (-0.9, -0.2)	0.01	-	-
Paternal education: Attended postsecondary (reference: less than postsecondary)	-1.7 (-2.7, -0.7)	≤0.001	-	-
Gestational diabetes: Yes	1.1 (0.4, 1.8)	≤0.001	8.8 (2.6, 30.0)	≤0.001
Parenting stress at age 5 using the PSI-SF Scale	0.1 (0.1, 0.1)	≤0.001	1.2 (1.1, 1.3)	≤0.001
Constant	50.0 (49.4, 50.5)	≤0.001	0.5^2^ (0.2^2^, 0.2^1^)	≤0.001

Caption: SDB = Sleep Disordered Breathing, based on 6 items; PSI-SF = Parenting Stress Index-Self Report, higher score presents increased levels of parenting stress; CES-D = Centre for Epidemiological Studies—Depression, higher scores represent increased maternal symptoms of depression

#### CBCL internalizing results multiple linear regression analyses (secondary outcome)

Screen-time above the two-hours/day threshold exposure at five years was associated with a 1.8-point higher internalizing behavior T-scores (95%CI: 0.6, 3.1 *p*≤0.001) after adjusting for physical activity, parent reported SDB symptoms, SES, breastfeeding, parenting stress, and maternal depression (model 1, Table 5). None of the screen-time thresholds were associated with clinically significant internalizing morbidity (model 2, Table 5).

**Table 5:** Multiple regression analysis examining associations between screen-time and the CBCL internalizing behavior problem T-score at five-years of age (n = 2,427).

	Model 1: Linear score Internalizing T-score		Model 2: Clinical cut-off Internalizing T-score ≥65	
Factor	Coefficient 95%CI	p-value	Coefficient 95%CI	p-value
**Screen-time at 5 years**:	Reference		Reference	
Between 30-minutes and 2 hours daily	0.7 (-0.1, 1.6)	0.10	1.0 (0.5, 2.1)	0.96
More than 2-hours daily	1.7 (0.4, 2.9)	≤0.001	1.4 (0.6, 3.4)	0.48
**Organized physical activity at 5 years**: More than 2-hours/week: yes	-1.4 (-2.1, -0.7)	≤0.001	-	-
**Parent-reported SDB symptoms at age 5**: Yes	2.3 (1.0, 3.6)	≤0.001	-	-
**SES: Family income ≥ $60,000 income** (reference: < $60,000 / year)	-1.2 (-2.3, -0.1)	0.03	0.4 (0.2, 0.7)	≤0.001
**Breastfeeding at 6 months** Yes	-0.9 (-1.8, -0.1)	0.04	-	-
**Parenting stress at 5 years using the PSI-SF Scal**e	0.3 (0.2, 0.3)	≤0.001	1.1 (1.1, 1.2)	≤0.001
**Maternal depression at 5 years using the CES-D Scale**	0.2 (0.1, 0.3)	≤0.001	-	-
Constant	41.5 (39.6, 43.5	≤0.001	0.7^4^ (0.8^5^, 0.8^3^)	≤0.001

Caption: SDB = Sleep Disordered Breathing, based on 6 items; SES = socioeconomic status; PSI-SF = Parenting Stress Index-Self Report, higher score presents increased levels of parenting stress; CES-D = Centre for Epidemiological Studies—Depression, higher scores represent increased maternal symptoms of depression

## Discussion

This analysis of data from a large-population based birth cohort demonstrated a dose-response relationship between screen-time and behavioral problems. Screen use was common in our sample of pre-school children with an average of 1·4 hours/day. Critically, screen-time above the two-hours threshold was associated with an increased risk of clinically significant externalizing morbidity and specifically inattention problems. The association between screen-time and behavioral morbidity was greater than any other risk factors including sleep duration, parenting stress, and socio-economic factors. Consistent with prior reports, we found that children who participated in more than two-hours/week of organized physical activity were less likely to experience mental health morbidity[31].

Results from this study may help clarify mixed findings from prior research. Two prior studies in pre-school children did not find an association between screen-time and behavior problems. A UK study reported that screen-time was not a risk factor for increased behavior problems among five-year old children[31]. That study[31] used a behavior screening questionnaire (Strength and Difficulties Questionnaire subscales) that may not be sensitive to clinically significant behavioral problems[32]. Alternatively, the inclusion of children with 30-minutes to two-hours screen-time in their reference category may have attenuated their findings. A small cross-sectional study[33] of 200 children ages two to five-years found no statistically significant association between increased screen-time and externalizing behavior problems in adjusted analysis[33]. Externalizing behavior problems were relatively more common among boys compared to girls in our sample. However, we did not observe a significant interaction between gender and screen-time exposure. Our findings confirm the results of similar studies showing associations between early television viewing and ADHD symptoms[18, 34, 35].

Behavioral problems identified in this study are unlikely to reflect a referral bias as CHILD study families were recruited from the general population. We used an empirically validated outcome to measure behavior and mental health problems in young children. The large sample size allowed us to observe associations between screen-time and clinically significant behavioral morbidity while controlling for multiple confounders.

### Limitations

One of the limitations of this current study is that screen-time, sleep, and physical activity, were parent-reported and not validated against objective measures. We only had repeated screen-time and behavior data available for a sub-sample of our cohort (n = 367) which limited our ability to determine directionality. As such, it is possible parents may respond to children who exhibit externalizing behavior difficulties by offering more screen-time or using increased opportunity for screen-time as a self-soothing strategy. Although we identified prior studies in school-aged children[18, 34, 36, 37] that have shown a significant association between screen-time and inattention while controlling for earlier attention problems. We were also not able to determine if the media content (e.g. educational, video gaming, social media), or screen type (television, computer, tablet) were important predictors of behavioral morbidity. For example, the UK Millennium Cohort Study[20] showed that television exposure (but not gaming) above three-hours/day at age five was significantly associated with conduct behavior problems by age seven years. The sample of 2,447 children included in our analysis, overall, represented a higher SES, an older maternal age, were more likely to be of Caucasian compared to the general population[38]. As a result, our findings may not be generalizable to other populations and should be replicated. Over 84% of our sample met the recommended ten to thirteen-hours of sleep for a preschool child[39, 40] limiting our ability to examine the impact of sleep duration on behavior.

### Future directions

Our results suggest that physicians and educators promote limiting young children’s screen-time exposure in line with recommended guidelines[22, 41, 42]. Future studies should include randomized controlled trials of healthy screen-time use to ascertain whether limiting children’s screen-time leads to differences in attention problems. It is possible that screen-time may increase with age and even fewer children will meet the recommended two-hours of screen-time/day. Future longitudinal studies should examine whether early exposure to electronic devices has potential negative effects for mental health through school-age and adolescence. The introduction of technology in the classroom warrants further investigation into associations between longer duration of screen-time exposure and behavioral development. Little is known about how the type of media consumed is linked to mental health outcomes, and whether screen content itself is detrimental or is a marker for less activity or social interaction. Increased use of video and text chat, social-media platforms, and social-apps may have differential effects on mental health outcomes in children.

## Conclusion

We provide results from one of the largest birth cohort studies to examine screen-time exposure and behavioral morbidity in pre-school children. Screen-time above the two-hours threshold at 5-years was associated with an increased risk of clinically relevant externalizing morbidity and specifically inattention problems. The association between screen-time and behavioral morbidity was greater than any other risk factor including sleep, parenting stress, and socio-economic factors. Our findings indicate that pre-school may be a critical period for supporting parents and families on education about limiting screen-time and supporting physical activity.

## Supporting information

S1 File List of CHILD study investigators contributors. (DOCX)

S2 File Description of supplemental methods. (DOCX)

S3 File Multivariate results for total behavior problems. (DOCX)

S1 Table Univariate t-test analysis results for associations between screen-time, physical activity, and sleep and behavioral morbidity at five years (*n* = 2322). Note: SD = standard deviation; SES: socioeconomic status; SDB = sleep disordered breathing ^a^Analyzed by One-way ANOVA *p≤0.05 based on Tukey post hoc test.
(DOCX)

S2 Table Univariate logistic regression results for associations between screen-time, physical activity, and sleep and behavioral morbidity. Caption: CI = confidence interval; SDB = sleep disordered breathing.
(DOCX)

S3 Table Univariate t-test analysis of associations between categorical explanatory variables and externalizing behavior problems (primary outcome) at five years (*n* = 2447). Note: SD = standard deviation; SES: socioeconomic status; SDB = sleep disordered breathing ^a^ Analyzed by One-way ANOVA *p≤0.05 based on Tukey post hoc test.
(DOCX)

S4 Table Univariate linear regression analysis of associations between continuous explanatory variables and externalizing behavior problems at five-years of age (*n* = 2,447). Caption: PSI-SF = Parenting Stress Index-Self Report, higher score presents increased levels of parenting stress; P-CDI = Parent-Child Dysfunction Index, higher scores reflect increased perceived difficulties; CES-D = Centre for Epidemiological Studies—Depression, higher scores represent increased maternal symptoms of depression.
(DOCX)

S5 Table Univariate t-test analysis of associations between categorical explanatory variables and internalizing behavior problems (primary outcome) at five years (*n* = 2447). Note: SD = standard deviation; SES: socioeconomic status; SDB = sleep disordered breathing a Analyzed by One-way ANOVA *p≤0.05 based on Tukey post hoc test.
(DOCX)

S6 Table Univariate linear regression analysis of associations between continuous explanatory variables and internalizing behavior problems at five-years of age (*n* = 2,447). Caption: PSI-SF = Parenting Stress Index-Self Report, higher score presents increased levels of parenting stress; P-CDI = Parent-Child Dysfunction Index, higher scores reflect increased perceived difficulties; CES-D = Centre for Epidemiological Studies–Depression, higher scores represent increased maternal symptoms of depression.
(DOCX)

S7 Table Univariate t-test analysis of associations between categorical explanatory variables and total behavior problems (primary outcome) at five years (*n* = 2447). Note: SD = standard deviation; SES: socioeconomic status; SDB = sleep disordered breathing a Analyzed by One-way ANOVA *p≤0.05 based on Tukey post hoc test.
(DOCX)

S8 Table Univariate linear regression analysis of associations between continuous explanatory variables and total behavior problems at five-years of age (n = 2,447). Caption: PSI-SF = Parenting Stress Index-Self Report, higher score presents increased levels of parenting stress; P-CDI = Parent-Child Dysfunction Index, higher scores reflect increased perceived difficulties; CES-D = Centre for Epidemiological Studies—Depression, higher scores represent increased maternal symptoms of depression.
(DOCX)

S9 Table Multiple regression analysis examining associations between screen-time and the CBCL total behavior problem T-score at five-years of age (n = 2,427). Caption: SDB = Sleep Disordered Breathing, based on 6 items; PCD-I = Parent-Child Dysfunction Index, higher scores represents; PSI-SF = Parenting Stress Index-Self Report, higher score presents increased levels of parenting stress; CES-D = Centre for Epidemiological Studies—Depression, higher scores represent increased maternal symptoms of depression.
(DOCX)

## References

[1] Duch H, Fisher EM, Ensari I, Harrington A. Screen time use in children under 3 years old: a systematic review of correlates. Int J Behav Nutr Phys Act. 2013;10:102 10.1186/1479-5868-10-102 23967799 PMC3844496

[2] Hoyos Cillero I, Jago R. Systematic review of correlates of screen-viewing among young children. Prev Med. 2010;51(1):3–10. 10.1016/j.ypmed.2010.04.012 20417227

[3] Ferrari GLM, Pires C, Sole D, Matsudo V, Katzmarzyk PT, Fisberg M. Factors associated with objectively measured total sedentary time and screen time in children aged 9–11 years. J Pediatr (Rio J). 2018. 10.1016/j.jped.2017.12.003 29306718

[4] Nikkelen SW, Valkenburg PM, Huizinga M, Bushman BJ. Media use and ADHD-related behaviors in children and adolescents: A meta-analysis. Dev Psychol. 2014;50(9):2228–41. 24999762 10.1037/a0037318

[5] Poitras VJ, Gray CE, Janssen X, Aubert S, Carson V, Faulkner G, et al Systematic review of the relationships between sedentary behaviour and health indicators in the early years (0–4 years). BMC Public Health. 2017;17(Suppl 5):868 10.1186/s12889-017-4849-8 29219092 PMC5773886

[6] Tremblay MS, Carson V, Chaput JP. Introduction to the Canadian 24-Hour Movement Guidelines for Children and Youth: An Integration of Physical Activity, Sedentary Behaviour, and Sleep. Appl Physiol Nutr Metab. 2016;41(6 Suppl 3):iii–iv. 10.1139/apnm-2016-0203 27306430

[7] Tremblay MS, Chaput JP, Adamo KB, Aubert S, Barnes JD, Choquette L, et al Canadian 24-Hour Movement Guidelines for the Early Years (0–4 years): An Integration of Physical Activity, Sedentary Behaviour, and Sleep. BMC Public Health. 2017;17(Suppl 5):874 10.1186/s12889-017-4859-6 29219102 PMC5773896

[8] Chaput JP, Colley RC, Aubert S, Carson V, Janssen I, Roberts KC, et al Proportion of preschool-aged children meeting the Canadian 24-Hour Movement Guidelines and associations with adiposity: results from the Canadian Health Measures Survey. BMC Public Health. 2017;17(Suppl 5):829 10.1186/s12889-017-4854-y 29219075 PMC5773883

[9] Colley RC, Garriguet D, Adamo KB, Carson V, Janssen I, Timmons BW, et al Physical activity and sedentary behavior during the early years in Canada: a cross-sectional study. Int J Behav Nutr Phys Act. 2013;10:54 10.1186/1479-5868-10-54 23642258 PMC3655822

[10] Pujadas Botey A, Bayrampour H, Carson V, Vinturache A, Tough S. Adherence to Canadian physical activity and sedentary behaviour guidelines among children 2 to 13 years of age. Prev Med Rep. 2016;3:14–20. 10.1016/j.pmedr.2015.11.012 26844180 PMC4733064

[11] Vandewater EA, Rideout VJ, Wartella EA, Huang X, Lee JH, Shim MS. Digital childhood: electronic media and technology use among infants, toddlers, and preschoolers. Pediatrics. 2007;119(5):e1006–15. 10.1542/peds.2006-1804 17473074

[12] Cliff DP, McNeill J, Vella SA, Howard SJ, Santos R, Batterham M, et al Adherence to 24-Hour Movement Guidelines for the Early Years and associations with social-cognitive development among Australian preschool children. BMC Public Health. 2017;17(Suppl 5):857 10.1186/s12889-017-4858-7 29219104 PMC5773906

[13] Garriguet D, Carson V, Colley RC, Janssen I, Timmons BW, Tremblay MS. Physical activity and sedentary behaviour of Canadian children aged 3 to 5. Health Rep. 2016;27(9):14–23. 27655168

[14] Growing up in a digital world: benefits and risks. The Lancet Child & Adolescent Health. 2018;2(2):79. 30169237 10.1016/S2352-4642(18)30002-6

[15] Ozmert E, Toyran M, Yurdakok K. Behavioral correlates of television viewing in primary school children evaluated by the child behavior checklist. Arch Pediatr Adolesc Med. 2002;156(9):910–4. 12197799 10.1001/archpedi.156.9.910

[16] Levine EL, Waite MB. Television viewing and attentional abilities in fourth and fifth grade children. Journal of Applied Developmental Psychology. 2000;21(6):667–79.

[17] Page AS, Cooper AR, Griew P, Jago R. Children’s screen viewing is related to psychological difficulties irrespective of physical activity. Pediatrics. 2010;126(5):e1011–7. 10.1542/peds.2010-1154 20937661

[18] Landhuis CE, Poulton R, Welch D, Hancox RJ. Does childhood television viewing lead to attention problems in adolescence? Results from a prospective longitudinal study. Pediatrics. 2007;120(3):532–7. 10.1542/peds.2007-0978 17766526

[19] Suchert V, Pedersen A, Hanewinkel R, Isensee B. Relationship between attention-deficit/hyperactivity disorder and sedentary behavior in adolescence: a cross-sectional study. Atten Defic Hyperact Disord. 2017;9(4):213–8. 10.1007/s12402-017-0229-6 28378132

[20] Swing EL, Gentile DA, Anderson CA, Walsh DA. Television and video game exposure and the development of attention problems. Pediatrics. 2010;126(2):214–21. 10.1542/peds.2009-1508 20603258

[21] Erratum: Screen time and young children: Promoting health and development in a digital world. Paediatr Child Health. 2018;23(1):83. 10.1093/pch/pxx197 PMC5815095 29601612

[22] Council On C Media. Media and Young Minds. Pediatrics. 2016;138(5). 10.1542/peds.2016-2591 27940793

[23] Subbarao P, Anand SS, Becker AB, Befus AD, Brauer M, Brook JR, et al The Canadian Healthy Infant Longitudinal Development (CHILD) Study: examining developmental origins of allergy and asthma. Thorax. 2015;70(10):998–1000. 10.1136/thoraxjnl-2015-207246 26069286

[24] Takaro TK, Scott JA, Allen RW, Anand SS, Becker AB, Befus AD, et al The Canadian Healthy Infant Longitudinal Development (CHILD) birth cohort study: assessment of environmental exposures. J Expo Sci Environ Epidemiol. 2015;25(6):580–92. 10.1038/jes.2015.7 25805254 PMC4611361

[25] Tamana SK, Smithson L, Lau A, Mariasine J, Young R, Chikuma J, et al Parent-reported symptoms of sleep disordered breathing is associated with increased behavioral problems at 2 years of age: The Canadian Healthy Infant Longitudinal Development (CHILD) birth cohort study. Sleep. 2017. 10.1093/sleep/zsx177 PMC5806574 29099980

[26] Tremblay MS, Carson V, Chaput JP, Connor Gorber S, Dinh T, Duggan M, et al Canadian 24-Hour Movement Guidelines for Children and Youth: An Integration of Physical Activity, Sedentary Behaviour, and Sleep. Appl Physiol Nutr Metab. 2016;41(6 Suppl 3):S311–27. 10.1139/apnm-2016-0151 27306437

[27] Moreno MA, Chassiakos YR, Cross C, Hill D, Ameenuddin N, Radesky J, et al Media Use in School-Aged Children and Adolescents. Pediatrics. 2016;138(5). 10.1542/peds.2016-2592 27940794

[28] Achenbach TM, Ruffle TM. The Child Behavior Checklist and related forms for assessing behavioral/emotional problems and competencies. Pediatr Rev. 2000;21(8):265–71. 10922023 10.1542/pir.21-8-265

[29] Achenbach TM, Edelbrock C, Howell CT. Empirically based assessment of the behavioral/emotional problems of 2- and 3- year-old children. J Abnorm Child Psychol. 1987;15(4):629–50. 3437096 10.1007/BF00917246

[30] Aebi M, Winkler Metzke C, Steinhausen HC. Accuracy of the DSM-oriented attention problem scale of the child behavior checklist in diagnosing attention-deficit hyperactivity disorder. J Atten Disord. 2010;13(5):454–63. 10.1177/1087054708325739 19372495

[31] Griffiths LJ, Dowda M, Dezateux C, Pate R. Associations between sport and screen-entertainment with mental health problems in 5-year-old children. Int J Behav Nutr Phys Act. 2010;7:30 10.1186/1479-5868-7-30 20409310 PMC2867988

[32] Mieloo C, Raat H, van Oort F, Bevaart F, Vogel I, Donker M, et al Validity and reliability of the strengths and difficulties questionnaire in 5–6 year olds: differences by gender or by parental education? PLoS One. 2012;7(5):e36805 10.1371/journal.pone.0036805 22629332 PMC3356337

[33] Tansriratanawong S, Louthrenoo O, Chonchaiya W, Charnsil C. Screen viewing time and externalising problems in pre-school children in Northern Thailand. J Child Adolesc Ment Health. 2017;29(3):245–52. 10.2989/17280583.2017.1409226 29240545

[34] Stevens T, Barnard-Brak L, To Y. Television viewing and symptoms of inattention and hyperactivity across time the importance of research questions. Journal of Early Intervention. 2009;31:215–26.

[35] Miller CJ, Marks DJ, Miller SR, Berwid OG, Kera EC, Santra A, et al Brief report: Television viewing and risk for attention problems in preschool children. J Pediatr Psychol. 2007;32(4):448–52. 10.1093/jpepsy/jsl035 17012738

[36] Johnson JG, Cohen P, Kasen S, Brook JS. Extensive television viewing and the development of attention and learning difficulties during adolescence. Arch Pediatr Adolesc Med. 2007;161(5):480–6. 10.1001/archpedi.161.5.480 17485625

[37] Gentile DA, Swing EL, Lim CG, Khoo A. Video game playing, attention problems, and impulsiveness: Evidence of bidirectional causality. Psychology of Popular Media Culture. 2012;1:62–70.

[38] Maghera A, Kahlke P, Lau A, Zeng Y, Hoskins C, Corbett T, et al You are how you recruit: a cohort and randomized controlled trial of recruitment strategies. BMC Med Res Methodol. 2014;14:111 10.1186/1471-2288-14-111 25260762 PMC4190339

[39] Hirshkowitz M, Whiton K, Albert SM, Alessi C, Bruni O, DonCarlos L, et al National Sleep Foundation’s sleep time duration recommendations: methodology and results summary. Sleep Health. 2015;1(1):40–3. 10.1016/j.sleh.2014.12.010 29073412

[40] Paruthi S, Brooks LJ, D'Ambrosio C, Hall WA, Kotagal S, Lloyd RM, et al Recommended Amount of Sleep for Pediatric Populations: A Consensus Statement of the American Academy of Sleep Medicine. J Clin Sleep Med. 2016;12(6):785–6. 10.5664/jcsm.5866 27250809 PMC4877308

[41] Carson V, Barnes J, LeBlanc CMA, Moreau E, Tremblay MS. Increasing Canadian paediatricians’ awareness and use of the new Canadian Physical Activity and Sedentary Behaviour Guidelines for ages 0 to 17 years. Paediatr Child Health. 2017;22(1):17–22. 10.1093/pch/pxx006 29483790 PMC5819840

[42] Canadian Paediatric Society DHTFOO. Screen time and young children: Promoting health and development in a digital world. Paediatr Child Health. 2017;22(8):461–77. 10.1093/pch/pxx123 29601064 PMC5823000

