# Supplementary material for: Screen-time is associated with inattention problems in preschoolers: Results from the CHILD birth cohort study
Source: PLoS One. 2019 Apr 17;14(4):e0213995. doi: 10.1371/journal.pone.0213995 (PMC6469768; doi:10.1371/journal.pone.0213995)
Supplement: S1 File — (DOCX) [file pone.0213995.s001.docx]

**S1: CHILD Study investigators contributors:**

| **Author** | **Affiliation** | **Author** | **Affiliation** |
| --- | --- | --- | --- |
| ***Subbarao P, (Director)*** | *The Hospital for Sick Children* | ***Turvey SE***  ***(co-Director)*** | *University of British Columbia* |
| ***Anand SS*** | *McMaster University* | ***Azad MB*** | *University of Manitoba* |
| ***Becker AB*** | *University of Manitoba* | ***Befus AD*** | *University of Alberta* |
| ***Brauer M*** | *University of British Columbia* | ***Brook JR*** | *University of Toronto* |
| ***Chen E*** | *Northwestern University, Chicago* | ***Cyr MM*** | *McMaster University* |
| ***Daley D*** | *University of British Columbia* | ***Dell SD*** | *The Hospital for Sick Children* |
| ***Denburg JA*** | *McMaster University* | ***Duan QL*** | *Queen’s University* |
| ***Eiwegger T*** | *The Hospital for Sick Children* | ***Grasemann H*** | *The Hospital for Sick Children* |
| ***HayGlass KT*** | *University of Manitoba* | ***Hegele RG*** | *The Hospital for Sick Children* |
| ***Holness DL*** | *University of Toronto* | ***Hystad P*** | *Oregon State University* |
| ***Kobor MS*** | *University of British Columbia* | ***Kollmann TR*** | *University of British Columbia* |
| ***Kozyrskyj AL*** | *University of Alberta* | ***Laprise C*** | *Université du Québec à Chicoutimi* |
| ***Lou WYW*** | *University of Toronto* | ***Macri J*** | *McMaster University* |
| ***Mandhane PJ*** | *University of Alberta* | ***Miller GE*** | *Northwestern University, Chicago* |
| ***Miller GE*** | *Northwestern University, Chicago* | ***Moraes TJ*** | *The Hospital for Sick Children* |
| ***Paré PD*** | *University of British Columbia* | ***Ramsey CD*** | *University of Manitoba* |
| ***Ratjen F*** | *The Hospital for Sick Children* | ***Sandford AJ*** | *University of British Columbia* |
| ***Scott JA*** | *University of Toronto* | ***Scott J*** | *University of Toronto* |
| ***Sears MR*** | *McMaster University* | ***Silverman F*** | *University of Toronto* |
| ***Simons FE*** | *University of Manitoba* | ***Takaro TK*** | *Simon Fraser University* |
| ***Tebbutt SJ*** | *University of British Columbia* | ***To T*** | *The Hospital for Sick Children* |
