# Supplementary material for: Screen-time is associated with inattention problems in preschoolers: Results from the CHILD birth cohort study
Source: PLoS One. 2019 Apr 17;14(4):e0213995. doi: 10.1371/journal.pone.0213995 (PMC6469768; doi:10.1371/journal.pone.0213995)
Supplement: S2 File — (DOCX) [file pone.0213995.s002.docx]

**S2: Supplemental Methods**

**Assessment of potential covariates associated with either screen-time use or mental health.**

**Birth history:** The gender of the child, birth weight (kg), gestational age (in weeks), birth order. number of siblings at time of child’s birth and maternal age were extracted from hospital birth charts.

**Socioeconomic status:** Family income, marital status, and highest education achieved were obtained from questionnaires completed at five-years of age. Mothers reported on their family income and was categorized, using the highest reported annual family income, as below or above $60,000. Both mothers and fathers reported on their highest education achieved. Highest education level was categorized into post-secondary or less than post-secondary education for mothers and fathers separately.

**Maternal Marital status** when the child was aged five-years was grouped in two categories; married or common law, or separated, divorced, or widowed, based on self-report questionnaire.

**Ethnicity** was categorized as Caucasian or other. Child ethnicity was determined based on maternal and paternal reported ethnicity.

**Physical activity at age five-years**: Moderate to vigorous intensity physical activity was reported as the number of days/week that their child was somewhat physically active for at least 60-minutes/day according to the Canadian 24-hour Movement Guidelines for Young Children^24^.

Parents also reported on the number of hours/week their child spent in moderately vigorous physical activity *outside* of school including a) *organized* and b) *unorganized* sports, games, and other activities. Children were categorized into those with less than two-hours or more than two-hours for each of *organized* and *unorganized* physical activity/week based on the Canadian 24-hour Movement Guidelines for Young Children^24^.

**Sleep duration and sleep quality**: Sleep duration was assessed at five-years based on parent report of average night sleep time and wake-time in a 24-hour period. Children were categorized into those who slept less than or longer than ten hours in total based on the National Sleep Foundation and the American Academy of Sleep Medicine recommended guideline for pre-school aged children^28,29^.

Sleep disordered breathing (SDB) symptoms, from snoring to obstructive sleep apnea syndrome, were assessed at five-years using seven yes/no items from the Pediatric Sleep Questionnaire (PSQ) sleep-related breathing disorder (SRBD) subscale^30^. Each of the seven items included had an odds ratio of 9 or more for being associated with OSAS^30^. Children with more than one-third of affirmative answers on the full 22-item SRBD subscale are considered high risk for OSAS^30^. Children with three or more affirmative SRBD questions were considered to have SDB for this analysis.

**Maternal parenting stress**: The mother reported on perceived parenting stress at five-years using the Parenting Stress Index - Short Form (PSI-SF)^31^, which assesses how confident parents feel about their parenting abilities when faced with undue stress. The PSI-SF includes a validity defensive subscale to determine the extent to which a parent has attempted to provide answers to appear socially capable with their parenting styles.

**Maternal depression**: Mothers reported symptoms of depression at five-years using a self-administered questionnaire based on the Center for Epidemiological Studies-Depression (CES-D): a 20-item self-administered questionnaire. The CES-D has a maximum score of 60 with higher scores indicating greater depressive symptoms. A score of 16 or greater identifies individuals at risk for clinical depression.

**Gestational diabetes**: Information on gestational diabetes was recorded between 36 to 39 weeks based on maternal report.

**Breastfeeding status** was based on maternal report on a nutrition questionnaire completed at three, six, 12, and 24 months.

**Household smoke:** Mothers reported household smoke exposure during their five-years in clinic visit.

**Parent-child interaction:** Mothers completed the Parent-Child Dysfunctional Interaction (P-CDI), a 12-item self-reported questionnaire used to assess perceived quality and strength of the parent-child relationship, during the five-year visit. Scores range from 12 to 60 with higher scores indicate that parents perceive that their child does not meet expectations and that interactions with the child are not reinforcing.
