# Supplementary material for: Screen-time is associated with inattention problems in preschoolers: Results from the CHILD birth cohort study
Source: PLoS One. 2019 Apr 17;14(4):e0213995. doi: 10.1371/journal.pone.0213995 (PMC6469768; doi:10.1371/journal.pone.0213995)
Supplement: S3 File — (DOCX) [file pone.0213995.s003.docx]

**S3: Multivariate results for Total Behavior Problems (secondary outcome)**

**CBCL Total score multiple linear regression analyses**:

Children who watched more than 2-hours of screen-time/day had a 1.9-point increase in their total behavior problems score (95%CI: 0.8, 3.1, *p*≤0.001; model 1, table s4) compared to children who watched less than 30-minutes. Screen-time effects exceeded those of gender, parenting stress, maternal depression, parent-child interaction, and SES. Only SDB symptoms (2.5 points, 95%CI: 1.4, 3.7, *p*≤0.001) had greater adverse effects on total behavior problems. Participating in more than 2-hours of organized physical activity/week was associated with reduced total behavior problems (-1.1 points, 95%CI: -1.7, -0.4, *p*≤0.001).

**Clinically relevant behavior problems (CBCL≥65)**:

Children exposed to between 30-minutes and 2-hours of screen-time were 5 times more likely to report clinically significant *total* behavior problems (95%CI: 1.0, 25.2, *p*=0.05; model 2, table s4) compared to children exposed to less than 30-minutes of screen-time daily. Longer screen-time duration (more than 2-hours) was associated with increased total behavior problems (OR 5.1, 95%CI: 0.9, 29.3 *p*=0.07) although this did not reach statistical significance. None of the screen-time thresholds were associated with an increased risk of clinically significant *internalizing* problems (model 2, table s5).
