# Supplementary material for: Screen-time is associated with inattention problems in preschoolers: Results from the CHILD birth cohort study
Source: PLoS One. 2019 Apr 17;14(4):e0213995. doi: 10.1371/journal.pone.0213995 (PMC6469768; doi:10.1371/journal.pone.0213995)
Supplement: S1 Table — Note: SD = standard deviation; SES: socioeconomic status; SDB = sleep disordered breathing aAnalyzed by One-way ANOVA *p≤0.05 based on Tukey post hoc test. (DOCX) [file pone.0213995.s004.docx]

**S1 Table. Univariate t-test analysis results for associations between screen-time, physical activity, and sleep and behavioral morbidity at five years (*n*=2322).**

|  | **CBCL Composite Score** | | | | | | | |
| --- | --- | --- | --- | --- | --- | --- | --- | --- |
|  | **Total T-Score** |  | **Externalizing T-Score** | | | **Internalizing T-Score** | | |
|  | **Mean (SD)** | **p-value** | **Mean (SD)** | **p-value** | | **Mean (SD)** | **p-value** | **n** |
| **Screen-time at 5 years:** |  |  |  |  |  | |  |  |
| Reference: < 30-minutes daily | 39.6 (7.9) | - | 38.2 (8.6) | - | 43.1 (8.2) | | - | 519 |
| Between 30-minutes and 2 hours daily | 41.0 (8.8) | <0.001 | 39.4 (9.4) | 0.03 | 44.4 (9.0) | | <0.001 | 1403 |
| More than 2-hours daily | 44.0 (10.1) | <0.001 | 42.1 (10.8) | ≤0.001 | 46.2 (9.6) | | <0.001 | 290 |
| **Screen-time at 3 years:** |  |  |  |  |  | |  |  |
| Reference: < 30-minutes daily | 40.5 (8.5) | - | 39.3 (9.2) | - | 43.8 (8.9) | | - | 423 |
| Between 30-minutes and 2 hours daily | 40.1 (8.1) | ns | 38.5 (8.8) | ns | 43.6 (8.3) | | ns | 804 |
| More than 2-hours daily | 42.2 (9.5) | 0.01 | 40.4 (9.8) | ns | 45.3 (9.5) | | <0.001 | 844 |
| **Organized physical activity at 5 years**:    Less than 2-hours/week | 39.8 (8.1) |  | 40.2 (9.9) |  | 43.1 (8.5) | |  | 1370 |
| More than 2-hours/week | 41.7 (9.2) | <0.001 | 38.4 (8.5) | ≤0.001 | 45.0 (9.0) | | <0.001 | 821 |
| **Unorganized physical activity at 5 years**:  Less than 2-hours/week | 41.2 (9.0) |  | 38.7 (8.7) |  | 43.7 (8.6) | |  | 1629 |
| More than 2-hours/week | 40.2 (8.2) | 0.03 | 39.7 (9.6) | 0.03 | 44.4 (8.9) | | 0.10 | 553 |
| **Parent-reported SDB symptoms at 5 years**:  Yes | 44.5 (10.6) |  | 42.1 (11.0) |  | 47.2 (10.1) | |  | 185 |
| No | 40.7 (8.6) | <0.001 | 39.3 (9.3) | ≤0.001 | 44.1 (8.8) | | <0.001 | 2029 |
| **Sleep duration at 5 years** |  |  |  |  |  | |  |  |
| Slept less than 10-hours | 42.3 |  | 39.3 (9.3) |  | 44.6 | |  | 108 |
| Slept more than 10-hours | 40.8 | <0.001 | 39.3 (9.3) | ≤0.001 | 44.3 | | ns | 1878 |

Note: SD= standard deviation; SES: socioeconomic status; SDB = sleep disordered breathing ^a^Analyzed by One-way ANOVA **p*≤0.05 based on Tukey post hoc test
