# Supplementary material for: Screen-time is associated with inattention problems in preschoolers: Results from the CHILD birth cohort study
Source: PLoS One. 2019 Apr 17;14(4):e0213995. doi: 10.1371/journal.pone.0213995 (PMC6469768; doi:10.1371/journal.pone.0213995)
Supplement: S2 Table — Caption: CI = confidence interval; SDB = sleep disordered breathing. (DOCX) [file pone.0213995.s005.docx]

**S2 Table. Univariate logistic regression results for associations between screen-time, physical activity, and sleep and behavioral morbidity.**

|  | **CBCL Composite Score** | | | | | | | | | | |
| --- | --- | --- | --- | --- | --- | --- | --- | --- | --- | --- | --- |
|  | **Total Clinical cut-off ≥65**  **(*n*=31/2427)** | | | | **Externalizing Clinical cut-off ≥65**  **(*n*=26/2427)** | | | | **Internalizing Clinical cut-off ≥65**  **(*n*=52/2427)** | | |
| **Explanatory variable** | **Crude OR**  **95%CI** | **n** | **p-value** | **Crude OR**  **95%CI** | | **n** | **p-value** | **Crude OR**  **95%CI** | | **n** | **p-value** |
| **Screen-time at 5 years:** |  |  |  |  | |  |  |  | |  |  |
| Reference: < 30-minutes daily | Reference | 2 |  | Reference | | 2 |  | Reference | | 10 |  |
| Between 30-minutes and 2 hours daily | 3.7 (0.9, 16.1) | 20 | 0.08 | 2.8 (0.6, 12.3) | | 15 | 0.17 | 1.2 (0.6, 2.4) | | 31 | 0.70 |
| More than 2-hours daily | 8.3 (1.8, 38.6) | 9 | 0.01 | 8.3 (1.8, 38.6) | | 9 | 0.01 | 2.0 (0.8, 4.8) | | 11 | 0.12 |
| **Organized physical activity at 5 years**:  Less than 2-hours/week | Reference | 25 |  | Reference | |  |  | Reference | |  |  |
| More than 2-hours/week | 0.3 (0.1, 0.9) | 5 | 0.02 | 0.3 (0.1, 0.9) | | 4 | ≤0.01 | 0.5 (0.3, 1.0) | | 12 | ≤0.001 |
| **Parent-reported SDB symptoms at 5 years**:  Yes | 3.2 (1.3, 7.4) | 7 | 0.01 | 1.9 (0.7, 5.6) | | 4 | 0.20 | 2.0 (0.9, 4.3) | | 8 | 0.08 |
| **Sleep duration > 10-hours**:  Yes | 0.4 (0.2, 0.8) | 22 | 0.01 | 0.3 (0.1, 0.5) | | 16 | ≤0.01 | 1.0 (0.5, 2.1) | | 45 | 1.00 |

Caption: CI= confidence interval; SDB = sleep disordered breathing
