# Supplementary material for: Screen-time is associated with inattention problems in preschoolers: Results from the CHILD birth cohort study
Source: PLoS One. 2019 Apr 17;14(4):e0213995. doi: 10.1371/journal.pone.0213995 (PMC6469768; doi:10.1371/journal.pone.0213995)
Supplement: S3 Table — Note: SD = standard deviation; SES: socioeconomic status; SDB = sleep disordered breathing a Analyzed by One-way ANOVA *p≤0.05 based on Tukey post hoc test. (DOCX) [file pone.0213995.s006.docx]

**S3 Table. Univariate t-test analysis of associations between categorical explanatory variables and externalizing behavior problems (primary outcome) at five years (*n*=2447).**

| **Categorical factors** | **CBCL Externalizing T-Score** | | |
| --- | --- | --- | --- |
|  | **Mean (*SD*)** | ***N*** | **p-value** |
| **Gender** |  |  |  |
| Boys | 40.5 (9.8) | 1268 | ≤0.001 |
| Girls | 38.7 (9.0) | 1159 |  |
| **SES: Family income at 5 years clinic visit** |  |  |  |
| ≥ $60,000 | 39.1 (9.1) | 1986 | ≤0.001 |
| < $60,000 | 42.1 (11.2) | 321 |  |
| **SES: Maternal education** |  |  |  |
| Post-secondary or higher | 40.9 (8.7) | 2236 | ≤0.001 |
| Less than post-secondary | 43.9 (11.4) | 144 |  |
| **Maternal ethnicity** |  |  |  |
| Caucasian | 39.9 (9.5) | 1810 | 0.01 |
| Other | 38.7 (9.4) | 602 |  |
| **Child ethnicity** |  |  |  |
| Caucasian | 39.9 (9.6) | 1606 | 0.03 |
| Other | 40.0 (9.2) | 797 |  |
| **Marital status** |  |  |  |
| Married or common law | 39.3 (9.3) | 2167 | ≤0.001 |
| Separated, divorced, or widowed | 43.2 (10.5) | 143 |  |
| **Birth Term** |  |  |  |
| Late preterm | 40.0 (10.1) | 98 | 0.66 |
| Full term | 39.6 (9.4) | 2287 |  |
| **Birth Order: Second born** |  |  |  |
| First born | 39.9 (9.6) | 1231 | 0.16 |
| Subsequent born | 39.3 (9.3) | 1157 |  |
| **Gestational diabetes** |  |  |  |
| Yes | 41.5 (10.9) | 104 | 0.03 |
| No | 39.5 (9.4) | 2276 |  |
| **Household smoke at 5 years** |  |  |  |
| Yes | 42.5 (10.3) | 297 | ≤0.001 |
| No | 40.0 (9.2) | 1832 |  |
| **Breastfeeding 3 months** |  |  |  |
| Yes | 39.6 (9.3) | 1734 | 0.04 |
| No | 40.3 (9.8) | 216 |  |
| **Breastfeeding 6 months** |  |  |  |
| Yes | 39.3 (9.3) | 1601 | 0.09 |
| No | 40.1 (9.3) | 476 |  |
| **Breastfeeding 12 months** |  |  |  |
| Yes | 39.5 (9.3) | 995 | 0.82 |
| No | 41.2 (8.7) | 1135 |  |

Note: SD= standard deviation; SES: socioeconomic status; SDB = sleep disordered breathing ^a^ Analyzed by One-way ANOVA **p*≤0.05 based on Tukey post hoc test
