# Supplementary material for: Screen-time is associated with inattention problems in preschoolers: Results from the CHILD birth cohort study
Source: PLoS One. 2019 Apr 17;14(4):e0213995. doi: 10.1371/journal.pone.0213995 (PMC6469768; doi:10.1371/journal.pone.0213995)
Supplement: S5 Table — Note: SD = standard deviation; SES: socioeconomic status; SDB = sleep disordered breathing a Analyzed by One-way ANOVA *p≤0.05 based on Tukey post hoc test. (DOCX) [file pone.0213995.s008.docx]

**S5 Table. Univariate t-test analysis of associations between categorical explanatory variables and internalizing behavior problems (primary outcome) at five years (*n*=2447).**

| **Categorical factors** | **CBCL Internalizing T-Score** | | |
| --- | --- | --- | --- |
|  | **Mean (*SD*)** | ***N*** | **p-value** |
| **Gender** |  |  |  |
| Boys | 44.5 (9.1) | 1268 | ≤0.001 |
| Girls | 44.4 (8.8) | 1159 |  |
| **SES: Family income at 5 years clinic visit** |  |  |  |
| ≥ $60,000 | 39.1 (9.1) | 1986 | ≤0.001 |
| < $60,000 | 42.1 (11.2) | 321 |  |
| **SES: Maternal education** |  |  |  |
| Post-secondary or higher | 44.2 (8.7) | 2236 | ≤0.001 |
| Less than post-secondary | 46.8 (10.4) | 144 |  |
| **Maternal ethnicity** |  |  |  |
| Caucasian | 44.6 (8.9) | 1810 | 0.01 |
| Other | 44.0 (9.3) | 602 |  |
| **Child ethnicity** |  |  |  |
| Caucasian | 44.5 (8.7) | 1606 | 0.03 |
| Other | 44.5 (9.5) | 797 |  |
| **Marital status** |  |  |  |
| Married or common law | 44.3 (8.9) | 2,167 | ≤0.001 |
| Separated, divorced, or widowed | 46.4 (9.3) | 143 |  |
| **Birth Term** |  |  |  |
| Late preterm | 45.1 (8.7) | 98 | 0.46 |
| Full term | 44.4 (9.0) | 2287 |  |
| **Birth Order: Second born** |  |  |  |
| First born | 45.2 (9.1) | 1231 | ≤0.001 |
| Subsequent born | 43.7 (8.7) | 1157 |  |
| **Gestational diabetes** |  |  |  |
| Yes | 47.1 (10.5) | 104 | ≤0.001 |
| No | 44.4 (8.9) | 2276 |  |
| **Household smoke at 5 years** |  |  |  |
| Yes | 46.7 (9.7) | 297 | ≤0.001 |
| No | 43.9 (8.7) | 1832 |  |
| **Breastfeeding 3 months** |  |  |  |
| Yes | 44.4 (8.9) | 1734 | 0.04 |
| No | 45.7 (9.3) | 216 |  |
| **Breastfeeding 6 months** |  |  |  |
| Yes | 44.1 (8.7) | 1601 | 0.01 |
| No | 44.3 (9.3) | 476 |  |
| **Breastfeeding 12 months** |  |  |  |
| Yes | 44.1 (8.7) | 995 | 0.15 |
| No | 44.7 (8.6) | 1135 |  |

Note: SD= standard deviation; SES: socioeconomic status; SDB = sleep disordered breathing a Analyzed by One-way ANOVA *p≤0.05 based on Tukey post hoc test
