# Supplementary material for: Screen-time is associated with inattention problems in preschoolers: Results from the CHILD birth cohort study
Source: PLoS One. 2019 Apr 17;14(4):e0213995. doi: 10.1371/journal.pone.0213995 (PMC6469768; doi:10.1371/journal.pone.0213995)
Supplement: S8 Table — Caption: PSI-SF = Parenting Stress Index-Self Report, higher score presents increased levels of parenting stress; P-CDI = Parent-Child Dysfunction Index, higher scores reflect increased perceived difficulties; CES-D = Centre for Epidemiological Studies—Depression, higher scores represent increased maternal symptoms of depression. (DOCX) [file pone.0213995.s011.docx]

**S8 Table. Univariate linear regression analysis of associations between continuous explanatory variables and total behavior problems at five-years of age (n=2,447).**

|  | **CBCL Total T-Score** | | | |
| --- | --- | --- | --- | --- |
| **Continuous factors** | **Unadjusted Coefficient (95%CI)** | **Mean** | **p-value** | ***N*** |
| **Maternal age** | -0.12 (-0.20, -0.03) | 32.6 | 0.01 | 2427 |
| **Gestational age at delivery** | 0.09 (-0.20, 0.37) | 39.6 | 0.56 | 2385 |
| **Gestational weight at birth (grams)** | 0.64 (-0.16, 1.44) | 3.5 | 0.12 | 2364 |
| **Breastfeeding duration (between 3 to 30 months)** | -0.10 (-0.16, -0.02) | 10.0 | 0.01 | 2055 |
| **Maternal alcohol consumption during pregnancy** | -0.27 (-0.86, 0.31) | 0.2 | 0.36 | 2163 |
| **Days spent in physical activity at age 5 years** | -0.7 (-1.6, -0.1) | 3.8 | 0.10 | 2192 |
| **Parenting stress at 5 years using the PSI-SF Scale** | 0.45 (0.39, 0.50) | 13.0 | ≤0.001 | 2091 |
| **Parent-child interaction at 5 years using the P-CDI** | 0.83 (0.76, 0.90) | 16.3 | ≤0.001 | 2080 |
| **Maternal depression at 5 years using the CES-D Scale** | 0.39 (0.33, 0.44) | 10.5 | ≤0.001 | 2090 |

Caption: PSI-SF = Parenting Stress Index-Self Report, higher score presents increased levels of parenting stress; P-CDI = Parent-Child Dysfunction Index, higher scores reflect increased perceived difficulties; CES-D = Centre for Epidemiological Studies – Depression, higher scores represent increased maternal symptoms of depression.
