# Supplementary material for: Screen-time is associated with inattention problems in preschoolers: Results from the CHILD birth cohort study
Source: PLoS One. 2019 Apr 17;14(4):e0213995. doi: 10.1371/journal.pone.0213995 (PMC6469768; doi:10.1371/journal.pone.0213995)
Supplement: S9 Table — Caption: SDB = Sleep Disordered Breathing, based on 6 items; PCD-I = Parent-Child Dysfunction Index, higher scores represents; PSI-SF = Parenting Stress Index-Self Report, higher score presents increased levels of parenting stress; CES-D = Centre for Epidemiological Studies—Depression, higher scores represent increased maternal symptoms of depression. (DOCX) [file pone.0213995.s012.docx]

**S9 Table. Multiple regression analysis examining associations between screen-time and the CBCL total behavior problem T-score at five-years of age (n = 2,427).**

|  | **Model 1: Linear score**  **Total T-score**  **AIC: 16794.4** | | **Model 2: Clinical cut-off**  **Total T-score ≥65**  **AIC: 274.3** | |
| --- | --- | --- | --- | --- |
| **Explanatory variable** | **Coefficient**  **95%CI** | **p-value** | **Odds Ratio**  **95%CI** | **p-value** |
| **Screen-time at 5 years**:  Reference: Less than 30-minutes daily | Reference |  | Reference |  |
| Between 30-minutes and 2-hours daily | 0.9 (0.1, 1.6) | 0.03 | 5.0 (1.0, 25.2) | 0.05 |
| More than 2-hours daily | 1.9 (0.8, 3.1) | ≤0.001 | 5.1 (0.9, 29.3) | 0.07 |
| **Organized physically active at 5 years**:  More than 2-hours/week: Yes | -1.1  (-1.7, -0.4) | ≤0.001 | 0.3  (0.1, 1.0) | 0.05 |
| **Parent-reported SDB symptoms at 5 years**:  Yes | 2.5 (1.4, 3.7) | ≤0.001 | - | - |
| **Gender**: male | 0.9  (0.3, 1.5) | ≤0.001 | 4.6  (1.8, 11.4) | ≤0.001 |
| **Family income** ≥ **$60,000 annual income**  (Reference: <$60,000 annual income) | -1.6 (-2.7, -0.6) | ≤0.001 | - | - |
| **Paternal education**  Attended postsecondary: yes  (Reference: Less than postsecondary) | -1.7 (-2.7, -0.7) | ≤0.001 | - | - |
| **Subsequent born: yes** | -1.3 (-2.3, -0.3) | 0.01 | - | - |
| **Parent-child interaction at 5 years using the PCDI** | 0.6 (0.6, 0.7) | ≤0.001 | 1.2 (1.1, 1.3) | ≤0.001 |
| **Parenting stress at 5 years using the PSI-SF Scale** | 0.1 (0.1, 0.2) | ≤0.001 | 1.2 (1.1, 1.2) | ≤0.001 |
| **Maternal depression at 5 years using the CES-D Scale** | 0.1 (0.1, 0.2) | ≤0.001 | - | - |

Caption: SDB = Sleep Disordered Breathing, based on 6 items; PCD-I = Parent-Child Dysfunction Index, higher scores represents; PSI-SF=Parenting Stress Index-Self Report, higher score presents increased levels of parenting stress; CES-D = Centre for Epidemiological Studies – Depression, higher scores represent increased maternal symptoms of depression
